# Ultimate Full Contact: Fight Outcome Characterization Concerning Their Methods, Occurrence Times and Technical–Tactical Developments

**DOI:** 10.3390/ijerph17197094

**Published:** 2020-09-28

**Authors:** Fernando C. Loio Pinto, Henrique Neiva, Célia Nunes, Mário C. Marques, António C. Sousa, Daniel A. Marinho, Luís Branquinho, Ricardo Ferraz

**Affiliations:** 1 Department of Sport Sciences, University of Beira Interior, 6201-001 Covilhã, Portugal; fl.fernandoloio@gmail.com (F.C.L.P); henriquepn@gmail.com (H.N.); celian@ubi.pt (C.N.); antonio_carlossousa@hotmail.com (A.C.S.); d.marinho@gmail.com (D.A.M); luis_branquinho@outlook.pt (L.B.); ricardompferraz@gmail.com (R.F.); 2 Research Centre in Sports, Health and Human Development, CIDESD, 6200-001 Covilhã, Portugal; 3 Centre of Mathematics and Applications, CMA-UBI, 6201-001 Covilhã, Portugal

**Keywords:** combat sports, styles, skills, stand-up and ground fighting, submission, technical, knockout, KO, attack combinations, single attack, counter-attack

## Abstract

Fight analysis produces relevant technical–tactical information. However, this knowledge is limited in hybrid full-contact combat sports. Therefore, this study aimed to characterize the results of the fights’ outcomes through the winners at the World Ultimate Full Contact (WUFC) Championships between 2008 and 2017. Methods: 170 combats between senior male fighters (master class) from 38 countries were observed; all fight outcome methods, their occurrence times, inherent skills and their development forms were analyzed through frequencies, percentages, crosstabs and chi-square test, considering a Fisher’s exact value of *p* < 0.05. The fight outcome methods were, in decreasing order, as follows: submission; decision and technical knockout (TKO); knockout (KO); and doctor stoppage. Only 19.4% fights completed the regular time 10 min (600 s), and 68.8% fight outcomes occurred in the first 5 min (300 s). Chokes were more used than joint locks, primarily developed in single actions. Head punches and kicks were the skills most responsible for KO, developed more in combinations and counter-attacks, while TKO was always through combination attacks and mostly by ground and pound. Ground fighting is most effective. In stand-up fighting, combination attacks and counter-attack are most effective. It is important to increase the technical–tactical capacities and adjustable decision-making to perform the regular fight time.

## 1. Introduction

Ultimate Full Contact, promoted by the World Ultimate Full Contact (WUFC), is a combat art based on the technical–tactical actions and principles of martial arts/combat sports (i.e., pankration, taekwondo, Muay Thai, boxing, sambo, jiu-jitsu, wrestling, etc.) [1,2]. Thus, Ultimate Full Contact is characterized by being a hybrid combat sport with a very complex structure, developed in stand-up and/or ground fighting, and allowing striking and submission grappling skills [1,2,3]. The WUFC Championships have been taking place in Portugal since 1988, where many top fighters participate annually [1,2,4].

This is a competition with great technical–tactical dynamics (i.e., offensive, counteroffensive and defensive actions) developed under constant and full physical contact of intermittent intensity throughout the combat [1,3]. In fact, fights are developed under a context of high variability, tenacity and unpredictability, where the winner can be found by different methods (e.g., decision, submission, knockout (KO) or technical knockout (TKO)) [1,3]. These methods are common to pankration, free fight, shooto and mixed martial arts (MMA), which resulted from high technical variability (i.e., punches; kicks; knees; elbow strikes; and submission grappling actions like grabs, twists, takedowns, chokes and locks) [5].

Consequently, some authors [6] consider that the skills inherent to the different fight outcomes can be associated with contextualized practices; that is, when the fight outcomes are by T(KO) and submission, the fighters present higher values of striking and grappling actions during the ground fight [6], while in the fight outcomes by decision (i.e., split or unanimous), the fighters have higher values of striking actions during stand-up fighting [6]. In addition, the techniques (i.e., skills) with which the fighters end the fight (e.g., punches, kicks, chokes) characterize and categorize their styles [7]. Furthermore, the frequency of victories for specific actions reflects the level of versatility of their training [8]. In fact, the technical–tactical actions performed through the fight are related to the type of outcome of the fight [9,10,11,12].

Therefore, some studies have characterized the percentages of fight outcomes in different modalities, by gender (i.e., male and female) [6,13], fighters’ classes (i.e., amateurs and professionals) [6,8,10,14,15] and skills [9,10,12]. As a result, some factors have been identified for the different fight outcomes, e.g., specific fighting methods, physical conditioning, age, weighting, timing between fights, timing into the fight, fighting importance and technical efficiency [10,13,16,17]. In addition, through MMA studies, it is known that one-fifth of all wins by KO resulted from head punches in the first minute at the beginning of the round [18,19], and in judo, a significant number of matches end before the expiry of the normal fight time [17]. Other previous studies [20,21,22,23,24,25,26,27,28,29,30] have provided some relevant information for this investigation but do not relate the results to the type of fight outcomes. For example, in studies [20,21], related to the development of skills in boxing, it was determined that using boxing combinations is a characteristic of winners, highlighting the importance of punching more in combinations than alone to be more effective [20]. This was in line with a review study that highlighted triple-punch combinations and counter-punch combinations as conditions for winning in novice and elite boxing competitions [21]. Moreover, it is known that attack/counter-attack in several combat sports has been shown to be a determining factor in winning fights due to its greater effectiveness, calculated through the ratio between attack/counter-attacks and effective attacks/counter-attacks [20,22,23,24,25,26,27,28,29,30].

Beyond this referred knowledge about the relationship that exists between the fight outcome methods/techniques and styles, little is known about the fight outcome methods and their inherent skills concerning their times of occurrence and forms of development, and, thus, more research is needed. In addition, little is known in general about the fight outcomes in Ultimate Full Contact, and the results can be of extreme importance in terms of some relevant recommendations for coaches and fighters. Therefore, the main aim of the present study was to characterize the fight outcomes in Ultimate Full Contact, which can certainly be extrapolated to other, similar modalities (i.e., hybrid full contact combat sports). An aim of this study was to increase knowledge of tactical behavior, thus analyzing not only the percentages of the fight outcome methods (i.e., decision, submission, knockout KO, technical knockout TKO, and doctor stoppage (DS)) and the inherent technical–tactical skills (e.g., armbar, choke, head punch, high kick, ground and pound), but also how they were developed (i.e., single attack, combination attack, or counter-attack) and their times of occurrence in the fights. The following hypotheses were considered: most of fights end by decision; the fights’ outcomes by medical decision are high, due to serious injuries or impediments to continue the fight; the fights that end before the regular time (i.e., KO, TKO, or submission) take place before the first 5 min of the fight; the fights that end by submission are mostly a sequence of choke skills; the fights that end by KO are mostly a sequence of punch skills; the fights that end by TKO are mostly a sequence of ground strike skills; the fights that end by KO are mostly a sequence of counterattack skills; the fights that end by submission are mostly a sequence of single submission skills; and the fights that end by TKO are mostly a sequence of combination strike skills.

## 2. Materials and Methods

The present research is related to a case study regarding the WUFC World Ultimate Full Contact Championships. For this study, 170 recorded fights developed under professional rules between 340 senior male fighters, carried out between 2008 and 2017, were analyzed. All variables were analyzed statistically in order to accurately conclude the purposes of the study.

The variables in question were characterized according to WUFC rules and terminology: winners (W) and losers (L); fight times; fight outcome methods (i.e., decision, submission, knockout KO, technical knockout TKO and doctor stoppage DS); technical–tactical skills inherent to the fight outcome methods (i.e., armbar, rear-naked choke, guillotine choke, triangle choke, leg lock, arm-triangle choke, heel hook, keylock, spinning back kick, high kick, head punch, knee strike, ground and pound and stand-up strikes); and how the technical–tactical skills are developed (i.e., single attack, attack combinations and counter-attack).

It should be noted that other technical–tactical actions also allowed in the WUFC regulations were not reported in this study, as they were not found in the sample. In addition, the inclusion criteria predetermined the above appointed five combat outcome methods, so the results by disqualification and related to combats that finished in a “draw” or “no contest” were not included.

In relation to the meaning of the fight outcome methods, according to the WUFC rules [3]: winning by decision means that the fight reaches the end of the regular time, and the result is decided by the judges through points scored; winning by submission happens when one of the fighters taps out due to a submission technique applied by the opponent; winning by knockout (KO) happens when one of the fighters is unable to continue the fight due to having suffered a strike; winning by technical knockout (TKO) occurs when the referee considers that one of the fighters demonstrates technical inferiority due to their opponent’s strikes.

### 2.1. Subjects

Three hundred and forty senior male athletes (master class—minimum level of national champions or high rank in different combat sports or martial arts, aged ≥18, among all weight divisions) representing 38 countries, 170 (50.0%) winners (W) and 170 (50.0%) losers (L) were included. These fighters participated in the WUFC World Ultimate Full Contact Championships held in Portugal each year between 2008 and 2017. The fighters were selected because they are top-ranked in the WUFC according to the inclusion criteria [4].

### 2.2. Instruments and Procedures

As instruments of data collection, detailed observation grids with all quantitative and qualitative variables were used, built in the Microsoft Excel Office 365 software (Microsoft, Washington, United States). The information was obtained through the recorded fights, provided by the WUFC World Ultimate Full Contact.

All combat recordings were observed in detail, with the necessary pauses and repeats, and fight outcome methods, technical–tactical actions, their development and occurrence times in the fights were all recorded. Then, the data were encoded and exported to the IBM SPSS Statistics 24 software (IBM, New York, NY, USA), where the respective statistical analyses were performed.

### 2.3. Statistical Analysis

Data analysis was performed using the IBM SPSS Statistics 24 software, where frequencies, percentages and crosstabs were calculated, through which the fighting times were compared with fight outcome methods, including their inherent technical–tactical actions and their form of development. Statistical significance was calculated through the chi-square test, considering the Fisher’s exact value as *p* < 0.05.

The fight time variable was recoded into three time interval subgroups: the first subgroup from 0 s to 300 s; the second subgroup from 301 s to 599 s; and the third subgroup referring to the end of the regular time (600 s). The minimum duration time observed in the fighting was 10 s and the maximum, 600 s, referring to the total fight time, consisting of a ten-minute round (1 × 10 min).

## 3. Results

### 3.1. The Fight Outcome Characterization Regarding the Methods and Occurrence Times

The 170 fights analyzed resulted in a total time of 43,120 s (statistical average 253.65 and standard deviation 203.65), of which 33 (19.4%) fights came to an end at the regular time (600 s), that is, 19.4% fight outcomes by decision (Table 1). In contrast, 117 (68.8%) fights finished during the first 5 min (300 s), where 73 (42.9%) fight outcomes were by submission, 17 (10.0%) fight outcomes were by KO, 26 (15.3%) fight outcomes were by TKO and 1 (0.6%) fight outcome was by DS (Table 1). In addition, it was observed that 20 (11.8%) fights finished after 5 min (300 s) but before the regular time of the fight, of which 11 (6.5%) fight outcomes were by submission, 1 (0.6%) fight outcome was by KO, 7 (4.1%) fight outcomes were by TKO and 1 (0.6%) outcome was by DS (Table 1). In the chi-square test, Fisher’s exact value was considered (46.7% > 20%), observing the value of 154.77 and *p* = 0.00, which proves that there was a statistically significant difference between the variables (*p* < 0.05).

From the values presented, it is clear that there are few battles that complete the regular time, with only 19.4% fight outcomes being by decision (points scored). Most of the fights (68.8%) finished before 5 min (300 s). The submission method was the predominant fight outcome, with its highest percentage before the 300 s, followed by the decision and TKO methods, both with the same percentages, and last, by the KO method with regard to the TKO and KO methods—these mostly occurred during the first 300 s. Another method observed was the DS, with only two cases, which proves to be quite positive in terms of behaviors and regulations.

Overall, the results showed the following frequencies and percentages: 84 (49.4%) by submission, 33 (19.4%) by decision, 33 (19.4%) by TKO, 18 (10.6%) by KO and 2 (1.2%) by DS.

### 3.2. The Fight Outcomes’ Characterization in Terms of the Inherent Technical–Tactical Actions

From the technical–tactical actions that were at the origin of the different fight outcome methods, the following were observed (Table 2): At the submission level: 17 (10.0%) were by armbar, 29 (17.1%) by rear-naked choke, 15 (8.8%) by guillotine choke, 9 (5.3%) by triangle choke, 2 (1.2%) by leg lock, 7 (4.1%) by arm-triangle choke, 3 (1.8%) by heel hook and 2 (1.2%) by keylock.

At the KO level: two (1.2%) were by spinning back kick, two (1.2%) by high kick, nine (5.3%) by head punch, two (1.2%) by knee strike and three (1.8%) by ground and pound.

At the TKO level: 21 (12.4%) were by ground and pound and 12 (7.1%) by stand-up strikes. 

### 3.3. The Technical–Tactical Characterization in Terms of Development

From the technical–tactical actions observed in the fighting results, the following were observed (Table 3):

At the submission level: 60 (35.3%) actions were carried out in single attacks, 20 (11.8%) actions were carried out in combination attacks and 4 (2.4%) were carried out in counter-attacks. 

At the KO level: four (2.4%) actions were carried out in single attacks, seven (4.1%) actions were carried out in combination attacks and seven (4.1%) were carried out in counter-attacks.

At the TKO level: 0 (0.0%) actions were carried out in single attacks, 33 (19.4) actions were carried out in combination attacks and 0 (0.0%) actions were carried out in counter-attacks.

It becomes clear that the fight outcomes for submission happened predominantly through technical–tactical actions carried out in single attacks; the fight outcomes by KO happened predominantly through technical–tactical actions carried out in combinations and counter-attacks; and the fight outcomes by TKO always took place through technical–tactical actions carried out in combinations attacks.

It was found that the development of the total technical–tactical actions that were at the origin of the fight outcomes before the end of the regular time were characterized by 64 (37.6%) in single development, 60 (35.3%) in combination development and 11 (6.5%) in counter-attack development.

## 4. Discussion

The present study aimed to characterize the fight outcomes in the Ultimate Full Contact, based on the fight outcome methods, their occurrence times, inherent technical–tactical actions and their development forms.

Overall, the results revealed that the fight outcome by submission was the most used method, followed by decision and TKO, both with the same percentages, then by KO and lastly by DS. Only 19.4% of the fights completed the regular time. In the fight outcomes by submission, chokes were the most used skills, and in the fight outcomes by KO, these happened more through head punches and kick skills, respectively, and were predominantly performed in counter-attacks and combinations. Fight outcomes by TKO occurred more through the ground and pound skill.

Therefore, the descending occurrence of the respective fight outcome methods (i.e., submission, decision, TKO and KO, respectively) is in accordance with the Fightmatrix statistical database (worldwide independent and comprehensive fighter ranking system) [31]. Considering all fight outcomes by year between 2008 and 2017, the following were observed: total fights: 173,288; submission: 67,134 (0.39); decision: 41,985 (0.24); and (T) KOs: 61,395 (0.35). In contrast, another study showed the decision as the main fight outcome reported [12], and this is in line with the following studies: in 174 female MMA fights, 72.4% (*n* = 126) fight outcomes were by the decision of the judges, 11.4% (*n* = 20) by submission, and 16% (*n* = 28) by KO or TKO [6]; the results of an MMA study that considered 304 bouts between male fighters demonstrated (*n* = 210) fight outcomes by decision, (*n* = 40) fight outcomes by submission, and (*n* = 54) fight outcomes by KO/TKO [14]. However, different results have been observed in a previous study, which demonstrated that the majority of amateur male fights ended in KO (57.7%) [15]. These are differentiated results concerning the preponderance of the fight outcome methods, which can be a sequence of several factors, such as styles, gender, age, weight, physical condition, fight importance, time in the fight and time without competing [10,13,16]. However, the preponderance of fight outcomes in the present study was based on the inherent technical–tactical actions, where the highest outcome percentages were related to fighting skills on the ground (i.e., 17.1% by rear-naked choke, 12.4% by ground and pound and 10.0% by armbar). In fact, these results are supported by an MMA study, where the winners proved to be more successful in performing the takedown when taking the fight to the ground [5]. Thus, a significant number of fight outcomes occurred due to fighting skills on the ground, which makes this style an effective combat strategy [32]. In line with this, grappling has been shown to play a dominant role in MMA fights, with the higher ground fighting skills being related with fight outcomes by submission, its use decreasing the number of KOs/TKOs [10,14,33]. This means that the ability to perform the fight on the ground is an efficient strategy; that is, grappling submission proved to be a determinant style in the Ultimate Full Contact. This is because the frequencies of the fight outcomes are related to fight skills or styles [7,8,9,10,11,12].

In fact, few fights (19.4%) in the present study completed the regular time (i.e., fight outcomes by decision through points scored). This is in line with a study carried out in judo with 125 fights between men and 68 between women, which also concluded that a significant number of fights ended before the regular time [17]. Thus, the same author suggested that the fighters should maintain a fast rhythm until the end of regular time with technical–tactical attack efficiency to increase the probability of success [17]. This means that the fighters must control decision making for their actions according the regular fight time, regulating their rhythm and interventions of attack until they can complete it. Furthermore, in the present analysis, the majority of the fights (68.8%) ended before 5 min (300 s) by submission, TKO and KO, respectively. In fact, it is known that in MMA, one-fifth of all victories through knockout before the end of the regular time occur in the first minute from the beginning of a round [34]. In contrast, in regard to the frequency of judo dynamics in the time function, the lowest offensive activity was observed in the first minute of the fight (i.e., 0.17 with the lowest effectiveness 0.03) [24]. These distinct fight dynamics may be related to the specific characteristics of each modality, since in the MMA rules, like Ultimate Full Contact or pankration, a wider range of technical–tactical actions is allowed when compared to judo. It should be noted that, unlike judo, in hybrid full contact combat sports, strikes are allowed, and the KO can happen at any time [18,19]. However, in the aforementioned judo study, as well as in the present investigation, the offensive activity decreased after 270 min, and the effectiveness decreased sharply after 260 min [24]. Thus, it is understood that in combat sports in general, the activity and efficiency tend to decrease throughout the combat.

In the fight outcomes by submission, the chokes were the skills most used (i.e., rear-naked choke, guillotine choke and triangle choke, respectively), while the armbar was the most common joint lock skill. However, the fights were ended more by choke skills, these being more efficient than joint locks. Similar results were found in a previous study, which demonstrated that fight outcomes by submission resulted more from choke skills than from lock skills [14]. In fact, the chokes are submission skills that are applied to the neck, a vital anatomical zone that is more difficult to defend or resist than joint lock skills applied to the elbow, shoulder or knee. They interrupt the flow of blood to the brain and, in turn, its supply of oxygen, forcing the opponent to give up quickly (i.e., tap out). In the present study, these skills were developed mainly by single attacks, as also characterized by isolated skill in a previous study [5]. This means that submission techniques are usually developed in isolation/single form, since they are skills very dependent on body weight and therefore complex from a biomechanical point of view, making them difficult to perform and combine.

In the fight outcomes by KO, head punches and kicks were, respectively, the predominant skills. This is in line with another MMA study, which showed that one-fifth of all wins by knockout before the end of the regular time results were through head punches [34]. In addition, in the fight outcomes by TKO, the ground and pound was the most used skill, which is in line with a study in which in ground fighting, fighters using the ground and pound, which is associated with a dominant position on the ground, had a clear tactical and technical advantage over their opponent [34]. In addition, it was observed that the aforementioned skills inherent to the KO/TKO were developed mainly in combination actions and in counter-attacks; in fact, some studies mention these two fight dynamics as very efficient, although without relating them to the fight outcomes [20,21,22,23,24,25,26].

It should be noted that the lowest incidence of the fight outcomes was by DS, with only 0.6% before 300 s and 0.6% after 300 s. From this, it can be determined that serious injuries are scarce in the Ultimate Full Contact competitions, which may be due to the experience and training of fighters and coaches [1]. Furthermore, the WUFC principles and regulations advocate the physical integrity of athletes as being more important than the show [1].

## 5. Conclusions

This investigation into fight outcomes presents relevant recommendations for coaches and fighters. The fight outcome by submission was the most used method, followed by the decision and technical knockout methods, both with the same percentages. This was followed by knockout and lastly by doctor stoppage, the latter having an insignificant value.

Most of the fights (68.8%) ended before 5 min (300 s) through submission, TKO and KO, respectively, and only 19.4% of fights completed the overall time of 10 min (600 s), ending by decision.

The fight outcomes by submission were mostly due to choke skills, using the rear-naked choke, guillotine choke and triangle choke, respectively, while the armbar was the joint lock skill most used. Regarding the fight outcomes by KO, these occurred more through head punch and kick skills, respectively, while the fight outcomes by TKO occurred more through the ground and pound skills. In addition, it was observed that the above-mentioned submission techniques are predominantly carried out in single actions; the stoppage skills by KO (e.g., head punches and kicks) are predominantly carried out in counter-attacks and attack combinations, while the TKO is the result of skills always carried out in attack combinations.

Based on the results found, coaches will be able to develop and improve the fighters‘ combat performance. It will also be interesting in future studies to analyze fight outcome characterization in women athletes and other competitive categories.

### Practical Suggestions

Based on the results found, and in order to develop and improve fighters’ performance, the following are suggested:

Develop ground fighting skills due to their higher effectiveness;

Stand-up strikers must develop skills that help them avoid being taken down (e.g., sprawl strategies);

Bearing in mind that (68.8%) fight outcomes are determined during the first 5 min (300 s) and only 19.4% fights complete the regular time 10 min (600 s), it is important to increase the technical–tactical capacities and adjustable decision making to meet the regular/overall fight time;

Develop attack combinations and counter-attack actions;

Develop the chokes and ground and pound skills, but also their defense methods, since these skills are highly effective in ground fighting;

Develop technical–tactical actions and fighting strategies (attacks/counter-attacks and respective defenses, e.g., head punching and dodging), taking into account the preponderance of the fight outcomes by submission and T/KO.

## Figures and Tables

**Table 1:** Fight outcome characterization regarding the methods and occurrence times.

Outcomes	Regular Time	Before 5 min		After 5 min	
By decision	33 (19.4%)				
By Submission		117 (68.8%)	73 (42.9%)	20 (11.8%)	11(6.5%)
By KO			17(10%)		1(0.6%)
By TKO			26(15.3%)		7(4.1%)
By DS			0.6%		1(0.6%)

Legend: KO = knockout; TKO = technical knockout; DS= doctor stoppage.

**Table 2:** Fight outcomes’ characterization in terms of the inherent technical–tactical actions.

Outcomes	Submission Level	KO Level	TKO Level
By armbar	17 (10%)		
By rear-naked	29 (17.1%)		
Guillotine choke	15 (8.8%)		
Triangle choke	9 (5.3%)		
Leg look	2 (1.2%)		
Arm-triangle choke	7 (4.1%)		
Heel hook	3 (1.8%)		
Keylock	2 (1.2%)		
Spinning Back Kick		2 (1.2%)	
High Kick		2 (1.2%)	
Head Punch		9 (5.3%)	
Knee Strike		2 (1.2%)	
Ground and pound		3 (1.8%)	21 (12.4%)
Stand-up Strikes			12 (7.1%)

Legend: KO = knockout; TKO= technical knockout.

**Table 3:** Technical–tactical characterization in terms of their development.

Outcomes	Submission Level	KO Level	TKO Level
Single Attacks	60 (35.3%)	4 (2.4%)	0 (0%)
Combinations Attacks	20 (11.8%)	7 (4.1%)	33 (19.4%)
Counter-attacks	4 (2.4 %)	7 (4.1 %)	0 (0%)
